# Supplementary material for: Antioxidant and Hepatoprotective Potential of Echinops ritro L. Extracts on Induced Oxidative Stress In Vitro/In Vivo
Source: Int J Mol Sci. 2023 Jun 10;24(12):9999. doi: 10.3390/ijms24129999 (PMC10298086; doi:10.3390/ijms24129999)
Supplement: Supplementary file 1 [file ijms-24-09999-s001.zip › ijms-2409616-supplementary.pdf]

Antioxidant and Hepatoprotective Potential of *Echinops ritro* L. Extracts on Induced Oxidative Stress In Vitro/In Vivo

Dimitrina Zheleva-Dimitrova [1,*,†], Rumyana Simeonova [2,*,†], Magdalena Kondeva-Burdina [2], Yonko Savov [3], Vessela Balabanova [1], Gokhan Zengin [4], Alexandra Petrova [2] and Reneta Gevrenova [1]

[1] Department of Pharmacognosy, Faculty of Pharmacy, Medical University-Sofia, 1000 Sofia, Bulgaria; vbalabanova@pharmfac.mu-sofia.bg (V.B.); rgevrenova@pharmfac.mu-sofia.bg (R.G.)
[2] Department of Pharmacology, Pharmacotherapy, and Toxicology, Faculty of Pharmacy, Medical University-Sofia, 1000 Sofia, Bulgaria; mkondeva@pharmfac.mu-sofia.bg (M.K.-B.); alexpetrova.work@gmail.com (A.P.)
[3] Institute of Emergency Medicine "N. I Pirogov", Bul. Totleben 21, 1606 Sofia, Bulgaria; yonko_savov@hotmail.com
[4] Physiology and Biochemistry Research Laboratory, Department of Biology, Science Faculty, Selcuk University, Campus, Konya 42130, Turkey; gokhanzengin@selcuk.edu.tr
* Correspondence: dzheleva@pharmfac.mu-sofia.bg (D.Z.-D.); rsimeonova@pharmfac.mu-sofia.bg (R.S.)
† These authors contributed equally to this work.

Supplementary material

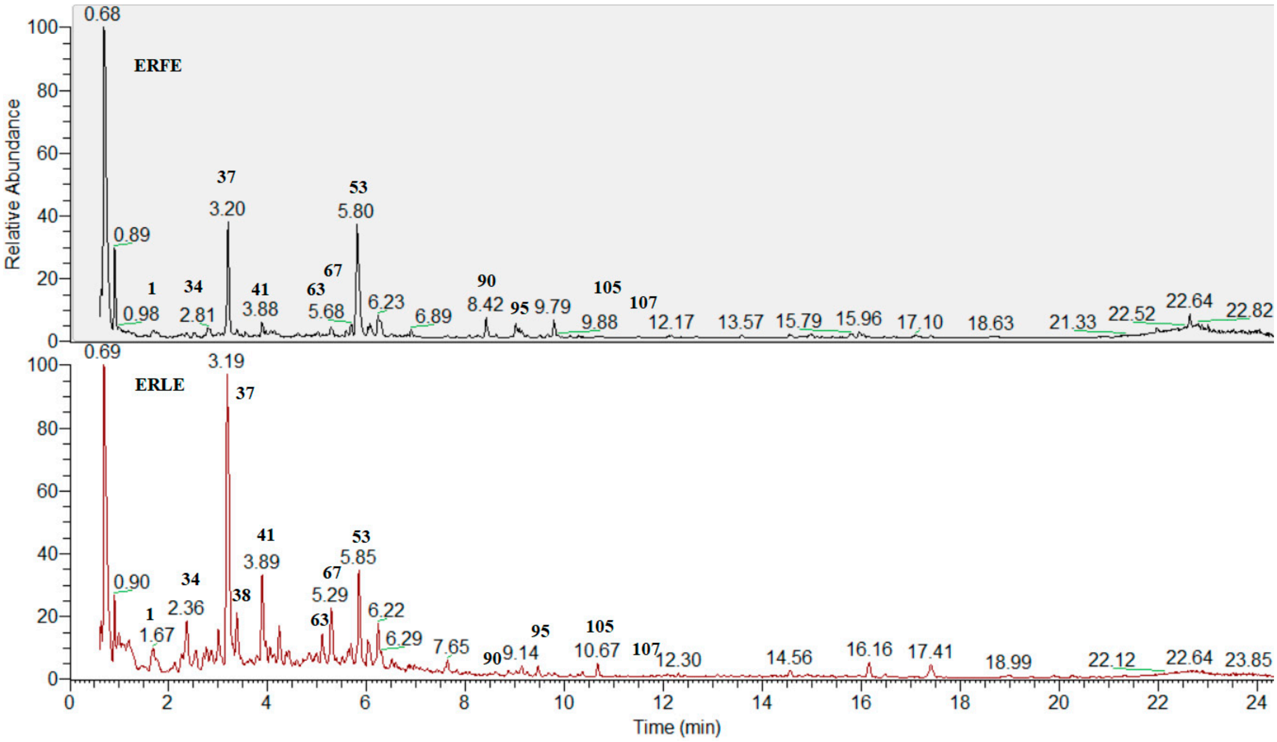

Figure S1. Total ion chromatograms in negative ion mode of the studied *Echinops ritro* extracts.

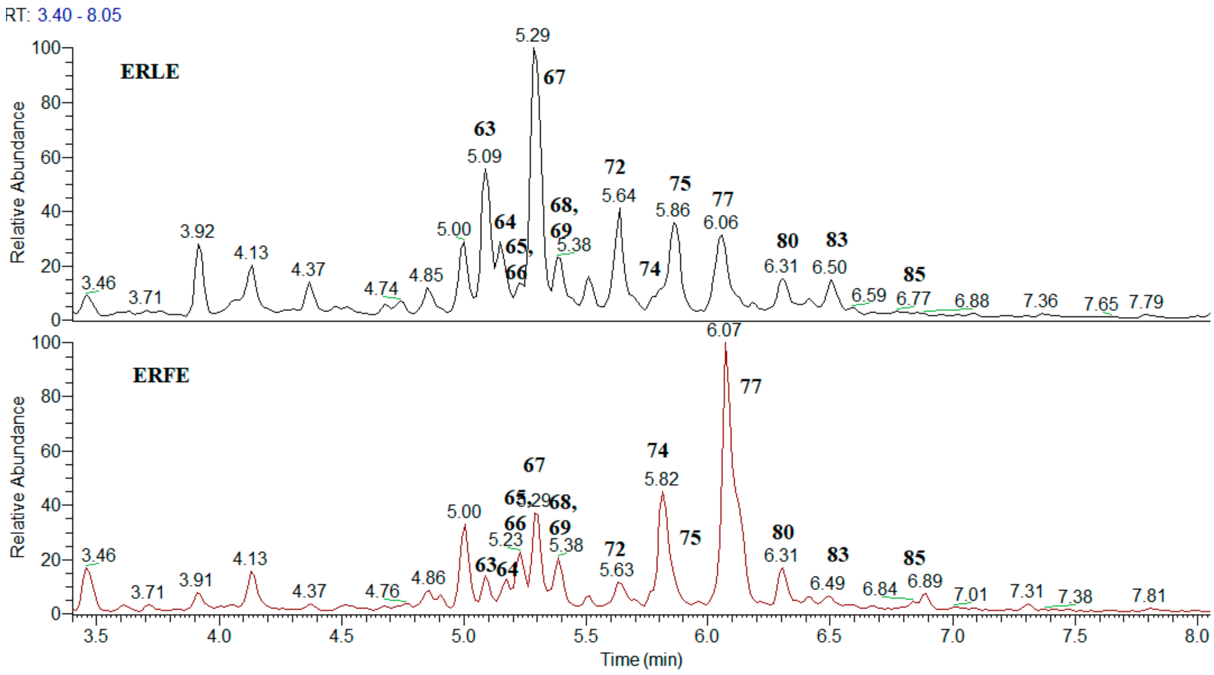

Figure S2. Extracted ion chromatograms in negative ion mode of flavonoid glycosides in *Echinops ritro* extracts.

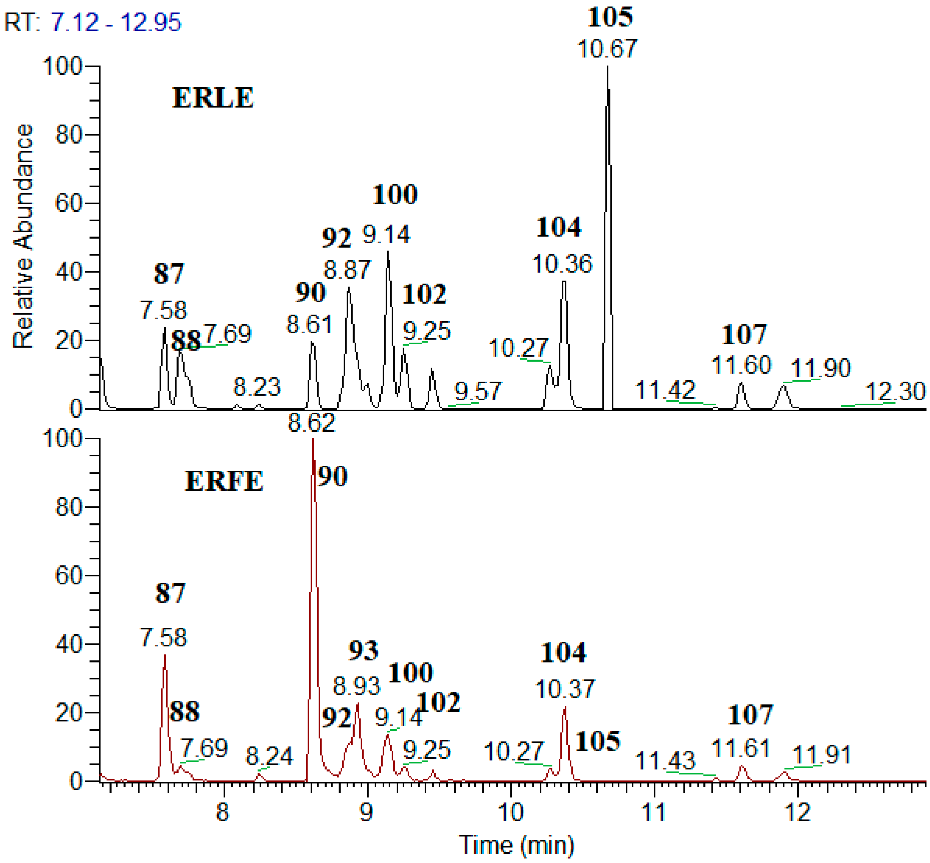

Figure S3. Extracted ion chromatograms in negative ion mode of flavonoid aglycons in *Echinops ritro* extracts.

Table S1. Secondary metabolites in the studied *Echinops ritro* extracts.

№	Identified/tentatively annotated compound	Molecular formula	Exact mass [M-H]$^-$	Fragmentation pattern in (-) ESI-MS/MS	t_R (min)	Δ ppm	Distribution
**Hydroxybenzoic, hydroxycinnamic acids, phenylethanoid glycosides, and coumarins**							
1.	protocatechuic acid-*O*-hexoside	$C_{13}H_{16}O_9$	315.0727	315.0726 (100), 153.0183 (26.9), 152.0103 (59.3), 123.0074 (2.8), 109.0286 (9.5), 108.0209 (90.1), 81.0329 (0.9)	1.67	1.316	A, B
2.	vanillic acid-*O*-hexoside[b]	$C_{14}H_{18}O_9$	329.0875	329.0877 (..),167.0340 (23.1), 152.0103 (100), 123.0438 (23.1), 108.0202 (53.5)	1.76	-0.381	A, B
3.	protocatechuic acid[a]	$C_7H_6O_4$	153.0181	153.0180 (16.99), 125.0230 (0.08), 109.0279 (100)	2.01	-8.247	A, B
4.	vanillyl alcohol-hexoside[b]	$C_{14}H_{20}O_8$	315.1085	315.1089 (4.1), 153.0545 (100), 123.0434 (54.9), 109.0280 (30.6)	2.12	1.299	A, B
5.	*p*-hydroxyphenylacetic acid *O*-hexoside[b]	$C_{14}H_{18}O_8$	313.0929	313.0941 (1.1), 151.0389 (100), 107.0488 (94.6)	2.13	3.926	A, B
6.	syringic acid-*O*-hexoside[b]	$C_{15}H_{20}O_{10}$	359.0985	359.0994 (9.67), 313.0929 (25.38), 197.0446 (100), 182.0210 (20.32), 123.0072 (39.99), 108.0201 (62.88)	2.29	0.473	A, B
7.	hydroxybenzoic acid-*O*-hexoside[b]	$C_{13}H_{16}O_8$	299.0778	299.0775 (12.9), 137.0231 (100), 109.0281 (0.7)	2.43	0.800	A, B
8.	*p*-hydroxyphenylacetic acid[b]	$C_8H_8O_3$	151.0401	151.0387 (100), 123.0436 (58.0), 121.0278 (77.2), 107.0486 (77.2),	2.50	-8.789	A, B
9.	gentisic acid-*O*-hexoside[b]	$C_{13}H_{16}O_9$	315.0727	315.0727 (37.3), 153.0181 (75.7), 135.0075 (5.1), 109.0280 (100), 91.0172 (0.5)	2.55	1.316	A, B
10.	syringic acid-*O*-pentoside[b]	$C_{14}H_{18}O_9$	329.0878	329.0882 (31.4), 197.0447 (100), 182.0212 (16.3), 167.0340 (14.0), 153.0545 (27.8), 138.0310 (17.0), 123.0073 (21.5)	2.69	1.199	A, B
11.	aesculetin-*O*-hexoside[b]	$C_{15}H_{15}O_9$	339.0724	339.0723 (24.7), 177.0182 (100), 149.0233 (1.2), 133.0281 (8.7), 105.0331 (0.2), 89.0381 (2.4),	2.71	0.515	A, B
12.	syringyl-*O*-hexose[b]	$C_{15}H_{20}O_{10}$	359.0984	359.0988 (100), 299.0772 (3.4), 269.0669 (1.1), 239.0557 (22.0), 197.0448 (38.0), 182.0207 (4.3), 153.0549 (3.7), 123.0073 (2.3)	2.76	2.952	A, B
13.	4-hydroxybenzoic acid[a,b]	$C_7H_6O_3$	137.0230	137.0229 (100), 119.0126 (1.3), 108.0201 (7.8), 93.0329 (11.3), 65.0380 (0.8)	2.84	-10.928	A, B
14.	hydroxybenzoic acid-*O*-hexoside isomer[b]	$C_{13}H_{16}O_8$	299.0778	299.0765 (1.4), 137.0230 (100), 93.0330 (49.4), 65.0380 (0.1)	3.00	-2.443	A, B

15.	*p*-hydroxyphenylacetic acid *O*-hexoside isomer[b]	$C_{14}H_{18}O_8$	313.0929	313.0941 (12.8), 151.0388 (100), 123.0073 (0.9), 109.0280 (3.6)	3.01	0.030	A, B
16.	*O*-caffeoyl hexose[b]	$C_{15}H_{18}O_9$	341.0871	341.0871 (16.02), 281.0667 (2.85), 251.0563 (6.58), 179.0338 (26.77), 173.0442 (100), 135.0436 (21.22)	3.08	-2.068	A, B
17.	quinic acid[b]	$C_7H_{12}O_6$	191.0549	191.0549 (100), 173.0444 (1.59), 127.0385 (4.12), 111.0435 (1.90)	3.15	-6.079	A, B
18.	*p*-hydroxyphenylacetic acid *O*-hexoside isomer[b]	$C_{14}H_{18}O_8$	313.0929	313.0945 (12.8), 151.0389 (10.5), 107.0487 (3.6)	3.30	0.030	B
19.	coumaric acid-*O*-hexoside[b]	$C_{15}H_{18}O_8$	325.0930	325.0924 (1.3), 163.0387 (53.5), 119.0486 (85.0)	3.33	-1.386	A, B
20.	*p*-coumaric acid[a, b]	$C_9H_8O_3$	163.0389	163.0390 (6.0), 135.0430 (0.2), 119.0448 (100)	3.35	-6.547	A, B
21.	shikimic acid[b]	$C_7H_{10}O_5$	173.0455	173.0445 (100), 155.0338 (2.2) 137.0232 (2.0), 127.0386 (1.5), 111.0437 (0.8), 93.0330 (77.0)	3.37	-5.875	A, B
22.	aesculetin[b]	$C_9H_6O_4$	177.0193	177.0182 (100), 149.0233 (3.1), 133.0281 (20.8), 105.0331 (10.6), 89.0381 (7.4)	3.46	-6.225	A, B
23.	vanillyl alcohol- (acetyl)-hexoside[b]	$C_{16}H_{22}O_9$	357.1191	357.1204 (4.6), 195.0656 (1.9), 153.0545 (100), 123.0437 (50.4)	3.47	3.681	A, B
24.	caffeic acid[a, b]	$C_9H_8O_4$	179.0339	179.0339 (21.95), 135.0437 (100), 107.0488 (2.07)	3.55	-6.211	A, B
25.	hydroxybenzoic acid-dihexoside[b]	$C_{19}H_{26}O_{13}$	461.1301	461.1299 (17.2), 137.0230 (100), 93.0331 (72.1)	3.63	-0.399	A, B
26.	ferulic acid[a, b]	$C_{10}H_{10}O_4$	193.0494	193.0494 (32.14), 178.0260 (73.72), 149.0593 (28.16), 134.0358 (100)	3.79	-6.330	A, B
27.	gentisic acid[a, b]	$C_7H_6O_4$	153.0180	153.0180 (80.68), 135.0072 (31.44), 109.0279 (100), 91.0175 (5.54)	3.86	-8.443	A, B
28.	*o*-coumaric acid[a, b]	$C_9H_8O_3$	163.0389	163.0388 (9.8), 135.0435 (0.9), 119.0487 (100)	4.56	-7.160	A
29.	vanillic acid[a]	$C_8H_8O_4$	167.0338	167.0338 (100), 152.0101 (28.20), 124.0150 (8.11), 108.0200 (24.59)	4.78	-7.376	A
30.	caffeic acid-(salicyl)-dihexoside[b]	$C_{28}H_{32}O_{16}$	623.1618	623.1629 (56.0), 485.1316 (2.7), 461.1096 (19.5), 323.0777 (4.0), 281.0656 (0.4), 341.0880 (19.2), 179.0340 (72.9), 137.0230 (100), 135.0437 (75.8), 93.030 (54.2),	5.78	1.833	A, B
31.	gentisic acid-(caffeoyl)-hexoside[b]	$C_{22}H_{22}O_{12}$	477.1038	477.1024 (69.4), 315.0732 (4.4), 153.0181 (100), 109.0280 (42.8), 179.0336 (3.3), 161.0233 (23.5), 135.0075 (10.2)	6.53	-2.996	A, B

32.	protocatechuic acid-(salicyl)-hexoside[b]	$C_{20}H_{20}O_{11}$	435.0933	435.0938 (100), 315.0724 (15.3), 297.0620 (9.2), 153.0183 (16.2), 152.0103 (37.0), 137.0231 (46.8), 109.0284 (6.2), 93.0331 (32.4)	7.65	1.208	A, B
**Mono-, diacyl- triacylquinic acids, and their hexosides**							
33.	1-caffeoylquinic acid[b]	$C_{16}H_{18}O_9$	353.0876	353.0875 (13.0), 191.0552 (100), 179.0339 (20.9), 173.0445 (3.1), 161.0238 (1.5), 135.0439 (14.6), 111.0439 (0.5), 93.0330 (3.6), 85.0279 (8.4)	1.89	-0.893	A, B
34.	neochlorogenic (3-caffeoylquinic) acid[a]	$C_{16}H_{18}O_9$	353.0867	353.0871 (39.93), 191.0551 (100), 179.0338 (57.43), 173.0444 (3.28), 135.0437 (43.91), 111.0437 (0.7), 93.0330 (5.4), 85.0279 (10.0)	2.36	-0.100	A, B
35.	caffeoylquinic acid-hexoside[b]	$C_{22}H_{27}O_{14}$	515.1395	515.1414 (100), 341.0872 (2.2), 323.0775 (53.0), 191.0553 (99.3), 179.0340 (5.5), 161.0233 (40.6), 133.0282 (10.6), 111.0435 (1.6), 93.0330 (6.1), 85.0279 (12.6), 135.0438 (5.9)	2.86	1.420	A, B
36.	3-*p*-coumaroylquinic acid[b]	$C_{16}H_{18}O_8$	337.0928	337.0920 (8.8), 191.0553 (15.5), 173.0444 (3.9), 163.0389 (100), 135.0431 (0.5), 119.0488 (28.2), 111.0434 (1.4), 93.0331 (2.8), 85.0280 (2.3)	3.02	-2.524	A, B
37.	chlorogenic (5-caffeoylquinic) acid[a]	$C_{16}H_{18}O_9$	353.0874	353.0875 (2.84), 191.0550 (100), 179.0336 (0.75), 161.0232 (1.40), 111.0435 (1.0), 93.0330 (2.9), 85.0279 (8.0)	3.19	-1.233	A, B
38.	4-caffeoylquinic acid[b]	$C_{16}H_{18}O_9$	353.0878	353.0882 (30.6), 191.0553 (48.8), 179.0340 (66.9), 173.0445 (100), 135.0437 (48.7), 127.0385 (2.2), 111.0434 (3.3), 93.0330 (19.5), 85.0279 (6.3)	3.37	-0.100	A, B
39.	3-feruloylquinic acid[b]	$C_{17}H_{20}O_9$	367.1040	367.1040 (20.79), 193.0497 (100), 191.0542 (1.61), 173.0446 (4.27), 134.0359 (47.02), 135.0396 (1.51)	3.44	1.402	A
40.	caffeoylquinic acid-hexoside[b]	$C_{22}H_{27}O_{14}$	515.1395	515.1414 (90.4), 341.0860 (0.6), 323.0775 (43.4), 191.0552 (100), 179.0342 (3.9), 173.0449 (1.6), 161.0233 (38.8), 135.037 (3.9), 133.0284 (9.9), 93.0329 (3.2), 85.0279 (11.6)	3.74	1.420	A, B
41.	5-caffeoylquinic acid isomer	$C_{16}H_{18}O_9$	353.0874	353.0883 (5.2), 191.0552 (100), 179.0339 (0.8), 173.0446 (0.7), 161.0233 (2.0), 127.0387 (1.5), 111.0436 (0.6), 93.0331 (2.3), 85.0279 (6.6)	3.89	1.543	A, B
42.	5-*p*-coumaroylquinic acid[b]	$C_{16}H_{18}O_8$	337.0928	337.0940 (10.33), 191.0551 (100), 173.0446 (6.06), 163.0385 (7.26), 119.0488 (5.51), 111.0438 (2.6), 85.0279 (4.9), 93.0330 (18.0)	3.95	-0.180	A, B
43.	3-caffeoyl-5-hydroxy-dihydrocaffeoylquinic acid[b]	$C_{25}H_{26}O_{13}$	533.1288	533.1306 (100), 371.0988 (12.9), 353.0883 (21.4), 335.0775 (6.2), 191.0553 (99.9), 179.0341 (54.2), 173.0445 (17.6), 161.0234 (7.2), 135.0438 (67.6), 93.0331 (8.2), 85.0280 (7.4)	4.02	1.043	A, B

44.	4-hydroxy-dihydrocaffeoyl-5-caffeoylquinic acid[b]	$C_{25}H_{26}O_{13}$	533.1288	533.1323 (13.60), 371.0988 (62.7), 353.0889 (3.1), 341.0892 (7.3), 335.0794 (1.8), 197.0448 (8.7), 191.0552 (21.7), 173.0445 (21.7), 161.0235 (4.3), 153.0542 (4.4), 135.0438 (40.4), 127.0390 (0.6), 111.0434 (3.1), 93.0331 (21.1), 85.0279 (7.7)	4.24	4.232	A, B
45.	5-feruloylquinic acid[b]	$C_{17}H_{20}O_9$	367.1034	367.1034 (18.32), 191.0551 (100), 173.0444 (23.75), 134.0358 (11.66), 111.0436 (3.63), 93.0330 (24.0), 85.0279 (4.0)	4.41	-0.096	A, B
46.	1-caffeoyl-3-hydroxy-dihydrocaffeoylquinic acid[b]	$C_{25}H_{26}O_{13}$	533.1288	533.1306 (34.5), 371.0987 (62.3), 353.0888 (5.1), 335.0784 (3.2), 191.0553 (14.5), 197.0451 (2.4), 179.0339 (13.1), 173.0445 (30.6), 161.0233 (4.8), 153.0544 (0.4), 135.0438 (100), 111.0436 (2.5), 93.0330 (7.5), 85.0278 (0.6)	4.43	1.043	A, B
47.	4-feruloylquinic acid[b]	$C_{17}H_{20}O_9$	367.1034	367.1039 (95.0), 193.0497 (12.7), 173.0446 (70.6), 134.0358 (23.6), 111.0438 (13.8), 93.0330 (100)	4.66	1.157	A, B
48.	dicaffeoylquinic acid-hexoside	$C_{31}H_{34}O_{17}$	677.1723	677.1740 (93.4), 515.1415 (72.9), 353.0884 (17.9), 341.0882 (26.4), 335.0776 (8.7), 323.0775 (17.6), 191.0553 (75.5), 179.0340 (91.9), 173.0444 (11.3), 161.0233 (25.6), 135.0438 (100), 111.0436 (2.4), 93.0331 (6.9), 85.0279 (6.4)	5.17	2.492	A, B
49.	dicaffeoylquinic acid-hexoside isomer	$C_{31}H_{34}O_{17}$	677.1723	677.1741 (100), 515.11266(22.6), 353.0883 (22.6), 341.0883 (8.0), 335.0776 (26.7), 323.0775 (4.8), 191.0552 (25.7), 179.0340 (57.6), 173.0445 (46.5), 161.0233 (30.0), 135.0438 (68.4), 111.0438 (4.0), 93.0331 (13.1), 85.0281 (2.7)	5.25	2.580	A, B
50.	1-hydroxy-dihydrocaffeoyl-3-caffeoylquinic acid[b]	$C_{25}H_{26}O_{13}$	533.1288	533.1310 (50.7), 371.0976 (1.1), 353.0883 (5.9), 335.0784 (3.2), 197.0442 (0.7), 191.0552 (100), 173.0445 (3.9), 135.0439 (12.5), 93.0330 (4.7), 85.0279 (8.4)	5.06	1.831	A, B
51.	dicaffeoylquinic acid-hexoside isomer[b]	$C_{31}H_{34}O_{17}$	677.1723	677.1740 (92.7), 515.1373 (52.8), 353.0883 (14.7), 341.0881 (52.6), 323.0781 (3.0), 191.0552 (15.7), 179.0340 (100), 173.0445 (61.0), 161.0232 (8.0), 135.0438 (93.0), 111.0436 (0.4), 93.0331 (23.4), 85.0279 (2.6)	5.66	2.403	B
52.	3,4-dicaffeoylquinic acid[a, b]	$C_{25}H_{24}O_{12}$	515.1190	515.1202 (100), 353.0883(14.4), 335.0775 (6.0), 191.0552 (29.5), 179.0340 (48.1), 173.0445 (58.9), 161.0232 (14.6), 135.0437 (47.6), 111.0430 (4.1), 93.0330 (13.3), 85.0279 (3.0)	5.69	1.321	A, B
53.	3,5-dicaffeoylquinic acid[a, b]	$C_{25}H_{24}O_{12}$	515.1189	515.1265 (15.2), 353.0883 (98.5), 335.0797 (0.4), 191.0553 (100), 179.0340 (52.0), 173.0443 (4.3), 161.0233 (4.4), 135.0437 (41.1), 111.0435 (1.5), 93.0328 (3.4), 85.0278 (6.3)	5.85	2.020	A, B

54.	3-caffeoyl-5-dehydrocaffeoylquinic acid[b]	$C_{25}H_{22}O_{12}$	513.1018	513.1018 (49.26), 351.0723 (100), 335.0771 (11.78), 191.0547 (20.51), 179.0343 (34.94), 161.0234 (9.90), 135.0437 (41.34), 133.0280 (78.71)	5.87	-3.974	A, B
55.	1,5-dicaffeoylquinic acid[a, b]	$C_{25}H_{24}O_{12}$	515.1190	515.1199 (33.7), 353.0882 (100), 335.0778 (3.1), 191.0552 (95.7), 179.0340 (54.5), 173.0447 (8.3), 161.0237 (6.2), 135.0438 (48.9), 93.0332 (4.3), 85.0280 (7.8)	6.03	0.720	A, B
56.	4,5-dicaffeoylquinic acid[a, b]	$C_{25}H_{24}O_{12}$	515.1190	515.1202 (100), 353.0882 (66.1), 335.0783 (1.0), 203.0339 (3.1), 191.05531 (34.8), 179.0340 (70.0), 173.0444 (97.3), 135.0438 (50.4), 111.0438 (3.0), 93.0330 (20.0), 85.0279 (3.2)	6.22	-1.163	A, B
57.	3-caffeoyl-5-*p*-coumaroylquinic acid[b]	$C_{25}H_{24}O_{11}$	499.1251	499.1253 (28.4), 353.0879 (54.8), 337.0929 (29.8), 191.0551 (100), 179.0345 (9.0), 173.0446 (16.4), 163.0390 (32.5), 161.0235 (7.8), 135.0439 (8.1), 119.0486 (21.4), 111.0435 (7.3), 93.0331 (13.9), 85.0278 (9.0)	6.53	1.513	A, B
58.	4-*p*-coumaroyl-5-caffeoylquinic acid[b]	$C_{25}H_{24}O_{11}$	499.1252	499.1243 (27.1), 353.0879 (0.7), 337.0933 (57.1), 191.0551 (4.1), 179.0336 (1.6), 173.0443 (100), 163.0388 (17.2), 161.0232 (1.7), 119.0487 (8.2), 111.0436 (3.3), 93.0329 (18.6)	6.92	-0.630	A, B
59.	4-feruloyl-5-caffeoylquinic acid[b]	$C_{26}H_{26}O_{12}$	529.1356	529.1349 (17.4), 367.1037 (69.1), 335.0703 (0.3), 193.0499 (16.6), 173.0445 (100), 166.0339 (4.3), 134.0360 (15.7), 111.0438 (3.2), 93.0331 (24.8), 85.0279 (0.8)	7.09	-0.490	A, B
60.	3,4,5-tricaffeoylquinic acid[a, b]	$C_{34}H_{30}O_{15}$	677.1528	677.1527 (100), 515.1191 (29.28), 353.0881 (40.46), 335.0777 (11.05), 191.0552 (38.25), 179.0339 (65.12), 173.0444 (81.58), 161.0231 (23.09), 135.0437 (78.63),	7.78	2.417	A, B
				**Flavonoids**			
61.	kaempferol *O*-dihexoside [b]	$C_{27}H_{30}O_{16}$	609.1464	609.1471 (100), 447.0926 (8.3), 285.0406 (32.1), 255.0298 (12.5), 227.0343 (3.5), 211.0397 (1.3), 163.0024 (8.1), 151.0022 (0.3), 135.0071 (2.4)	4.45	-1.629	A, B
62.	eriodyctiol *O*-dihexoside [b]	$C_{27}H_{32}O_{16}$	611.1618	611.1616 (9.9),449.1096 (100), 287.0563 (68.3), 151.0024 (53.7), 135.0438 (46.2), 107.0123 (11.0)	4.65	-0.324	A, B
63.	rutin [a,b]	$C_{27}H_{30}O_{16}$	609.1464	609.1474 (100), 301.0352 (37.8), 300.0279 (67.6), 271.0250 (31.0), 255.0302 (13.5), 243.0300 (6.5), 227.0342 (0.8), 215.0347 (0.5), 178.9977 (2.9), 163.0021 (0.6), 151.0024 (5.2), 121.0275 (0.3), 107.0122 (2.2)	5.09	2.121	A, B
64.	isoquercitrin [a]	$C_{21}H_{20}O_{12}$	463.0885	463.0890 (100), 301.0345 (17.1), 300.0278 (69.8), 271.0252 (0.7), 255.0294 (1.3), 243.0294 (1.4), 227.0340 (2.3)	5.15	0.585	A, B

65.	luteolin 7-*O*-rutinoside [a,b]	$C_{27}H_{30}O_{15}$	593.1512	593.1523 (92.0), 285.0406 (100), 256.0368 (0.7), 241.0478 (0.5), 217.0502 (0.5), 175.0390 (2.0), 151.0023 (4.4), 133.0282 (5.1), 107.0123 (2.3)	5.22	1.832	A, B
66.	patuletin *O*-rutinoside [b]	$C_{28}H_{32}O_{17}$	639.1567	639.1583 (100), 331.0458 (27.7), 330.0385 (57.0), 315.0152 (17.4), 287.0201 (17.9), 271.0255 (3.5), 259.0248 (4.1), 243.0296 (3.4), 231.0296 (3.2), 215.0344 (3.3), 203.0349 (1.2), 187.0393 (2.0), 165.9897 (3.9), 164.9819 (3.0), 136.9866 (0.5)	5.22	2.484	B
67.	hyperoside [a]	$C_{21}H_{20}O_{12}$	463.0885	463.0882 (11.25), 301.0349 (40.34), 300.0276 (80.42), 271.0247 (40.98), 255.0294 (16.40), 151.0247 (6.15), 121.0280 (1.12)	5.29	0.585	A, B
68.	luteolin-*O*-hexuronide [b]	$C_{21}H_{18}O_{12}$	461.0736	461.0729 (55.09), 285.0402 (100), 284.0317 (0.67), 267.0287 (0.27), 151.0022 (4.39), 133.0280 (8.67), 107.0122 (2.28)	5.37	0.718	A, B
69.	luteolin-7-*O*-glucoside [a,b]	$C_{21}H_{19}O_{11}$	447.0934	447.0934 (32.03), 285.0403 (100), 257.0452 (1.37), 151.0023 (8.18), 133.0280 (10.31), 107.0124 (3.54)	5.39	0.348	A, B
70.	patuletin *O*-hexoside [b]	$C_{22}H_{22}O_{13}$	493.0987	493.0996 (100), 331.0457 (31.9), 330.0385 (64.8), 315.0152 (21.3), 287.0200 (19.5), 271.0248 (4.0), 259.0251 (4.5), 243.0295 (3.7), 231.0298 (4.5), 215.0348 (3.6), 203.0342 (1.6), 187.0392 (2.7), 165.09898 (4.1), 164.9819 (3.1), 136.9868 (1.0)	5.45	1.655	A, B
71.	quercetin *O*-acetylhexoside [b]	$C_{23}H_{22}O_{13}$	505.0988	505.0999 (100), 463.0881 (0.8), 301.0351 (38.5), 300.0278 (92.0), 271.0250 (40.7), 255.0298 (18.3), 243.0295 (10.7), 227.0341 (3.5), 178.9980 (2.4), 151.0025 (5.8), 107.0121 (2.5)	5.60	2.210	A, B
72.	kaempferol 3-*O*-rutinoside [a,b]	$C_{27}H_{30}O_{15}$	593.1512	593.1522 (100), 285.0405 (69.4), 284.0328 (38.9), 255.0298 (28.3), 227.0345 (18.9), 211.0396 (1.5), 163.0028 (1.2), 151.0023 (1.5), 135.0073 (1.5), 107.0122 (2.1)	5.64	1.630	A, B
73.	isorhamnetin 3-*O*-rutinoside [a,b]	$C_{28}H_{32}O_{17}$	623.1618	623.1630 (100), 315.0504 (81.2), 300.0277 (16.0), 271.0249 (26.1), 255.0299 (9.5), 243.0298 (14.6), 227.0347 (3.1), 215.0345 (2.3), 199.0391 (3.3), 151.0026 (2.5), 107.0124 (0.5)	5.78	2.025	A, B
74.	apigenin *O*-rutinoside [b]	$C_{27}H_{30}O_{14}$	577.1563	577.1574 (37.4), 269.0455 (100), 225.0549 (1.2), 151.0024 (1.0), 117.0332 (3.7), 107.0123 (2.3)	5.81	1.960	A, B
75.	kaempferol-3-*O*-glucoside [a,b]	$C_{21}H_{19}O_{11}$	447.0934	447.0934 (100), 285.0398 (24.96), 284.0325 (53.68), 257.0452 (1.37), 151.0022 (1.93), 107.0120 (0.31)	5.86	0.348	A, B
76.	isorhamnetin 3-*O*-glucoside [a,b]	$C_{22}H_{21}O_{12}$	477.1044	477.1042 (100), 315.0504 (14.28), 314.0434 (48.91), 300.0264 (4.34), 299.0197 (6.38), 285.0404 (6.09), 271.0247 (22.71), 243.0294 (19.24), 215.0344 (4.04), 165.9895 (1.68), 151.0026 (2.26)	6.01	1.092	A, B

77.	apigenin 7-*O*-glucoside [a,b]	$C_{21}H_{19}O_{10}$	431.0980	431.0983 (100), 269.0448 (26.71), 268.0375 (40.3), 151.0024 (3.25), 117.0330 (1.78), 107.0122 (2.26)	6.07	-0.881	A, B
78.	apigenin *O*-hexuronide	$C_{21}H_{18}O_{11}$	445.0774	445.0780 (25.27), 269.0453 (100), 117.0329 (7.04), 151.0022 (2.31), 107.0122 (3.48)	6.12	-0.617	A, B
79.	kaempferol *O*-acetylhexoside [b]	$C_{23}H_{22}O_{12}$	489.1038	489.1046 (100), 285.0403 (67.4), 284.0328 (69.1), 255.0798 (48.0), 227.0346 (29.2), 211.0398 (2.6), 178.9957 (0.1), 163.0027 (1.4), 135.0074 (1.7), 107.0124 (2.1), 151.0025 (1.9)	6.29	1.433	A, B
80.	chrysoeriol *O*-hexoside [b]	$C_{22}H_{22}O_{11}$	461.1093	461.1098 (100), 446.0863 (18.8), 299.0559 (8. 2), 298.0485 (12.9), 284.0315 (5.8), 163.0029 (1.6), 283.0251 (20.5), 255.0299 (48.8), 227.0352 (1.4), 151.0025 (0.3), 117.0334 (1.7)	6.31	0.684	A, B
81.	chrysoeriol *O*-hexuronide [b]	$C_{22}H_{20}O_{12}$	475.0885	475.0885 (89.09), 300.0596 (6.89), 299.0560 (100), 285.0366 (5.85), 284.0325 (71.24), 151.0024 (2.77), 133.0123 (0.32), 117.0179 (2.20), 107.0117 (1.59)	6.33	0.381	A, B
82.	isorhamnetin *O*-acetylhexoside [b]	$C_{24}H_{24}O_{13}$	519.1144	519.1153 (100), 315.0512 (48.2), 314.0458 (59.9), 299.0202 (18.8), 271.0250 (25.0), 255.0301 (8.2), 243.0297 (20.2), 227.0340 (5.2), 199.0394 (6.1), 151.0026 (3.9), 107.0121 (0.9)	6.47	1.707	B
83.	jaceosidine O-hexoside [b]	$C_{23}H_{24}O_{12}$	491.1195	491.1204 (100), 329.0670 (4.9), 328.0589 (7.5), 314.0421 (7.6), 313.0358 (34.9), 298.0122 (10.6), 270.0172 (12.9), 242.0226 (2.5), 164.9821 (1.0), 163.0024 (2.2), 136.9872 (2.4), 147.0441 (2.0), 132.0196 (1.8)	6.50	1.875	A, B
84.	eupatorin *O*-hexoside [b]	$C_{24}H_{26}O_{12}$	505.1351	505.1362 (100), 343.0826 (39.7), 328.0592 (0.6), 313.0356 (12.3), 285.0404 (6.7), 257.0448 (2.4), 241.0501 (1.1), 226.0269 (0.4), 214.0261 (0.4), 198.0321 (1.7), 167.0343 (1.1), 153.0181 (1.2), 134.0362 (4.2), 139.0383 (0.6), 137.0235 (1.3)	6.67	2.139	A, B
85.	cirsimaritin *O*-hexoside [b]	$C_{23}H_{24}O_{11}$	475.1246	475.1252 (100), 313.0721 (63.6), 298.0484 (33.1), 283.0249 (14.2), 269.0452 (12.6), 255.0298 (29.4), 227.0344 (1.3), 164.9826 (0.2), 163.0027 (1.0), 119.0487 (2.9)	6.76	1.379	A, B
86.	luteolin *O*-acetylhexoside [b]	$C_{23}H_{22}O_{12}$	489.1038	489.1050 (21.5), 285.0405 (100), 267.0286 (0.2), 255.0298 (0.2), 241.0504 (1.8), 217.0499 (1.3), 199.0389 (2.4), 175.0391 (2.9), 151.0025 (5.9), 133.0281 (10.4), 107.0123 (2.5)	6.85	2.312	B
87.	luteolin [a,b]	$C_{15}H_{9}O_{7}$	285.0406	285.0405 (100), 151.0023 (1.72), 133.0283 (18.44), 107.0122 (2.47)	7.58	0.346	A, B
88.	patuletin [b]	$C_{16}H_{12}O_{8}$	331.0464	331.0463 (100), 316.0227 (54.8), 287.0197 (9.2), 271.0245 (4.7), 259.0253 (2.7), 243.0291 (2.1), 165.9897 (10.3), 139.0025 (6.6), 136.9864 (0.8), 121.0281 (2.0)	7.70	1.388	B

89.	apigenin O-acetylhexoside [b]	$C_{23}H_{22}O_{11}$	473.1089	473.1096 (100), 413.0849 (0.4), 269.0449 (16.3), 268.0378 (52.8), 151.0024 (2.9), 117.0328 (1.0), 107.0121 (2.0)	7.82	1.406	A, B
90.	apigenin [a,b]	$C_{15}H_9O_5$	269.0455	269.0453 (100), 151.0023 (6.03), 149.0230 (5.20), 117.0329 (20.52), 107.0122 (5.32)	8.61	-0.285	A, B
91.	hispidulin [b]	$C_{16}H_{12}O_6$	299.0561	299.0562 (57.2), 255.0297 (1.1), 227.0345 (2.8), 211.0389 (1.8), 165.9897 (0.4), 164.0106 (2.0), 136.9867 (13.6), 117.0331 (1.4)	8.84	0.430	A, B
92.	cirsiliol [b]	$C_{17}H_{14}O_7$	329.0677	329.0670 (100), 314.0436 (57.2), 299.0200 (34.5), 271.0249 (22.3), 255.0305 (0.3), 227.0347 (11.5), 215.0349 (2.1), 185.0235 (3.2), 161.0233 (11.3), 151.0022 (1.9)	8.87	1.045	A, B
93.	chrysoeriol [a,b]	$C_{16}H_{12}O_6$	299.0561	2299.0562 (100), 284.0327 (86.8), 256.0376 (18.7), 227.0346 (2.6), 211.0394 (1.1), 151.0025 (3.4), 107.0123 (2.8)	8.92	0.330	A, B
94.	spinacetin [b]	$C_{17}H_{14}O_8$	345.0616	345.0619 (100), 330.0384 (3.3), 315.0151 (23.5), 287.0199 (14.2), 271.0260 (1.2), 259.0247 (14.9), 243.0302 (1.1), 231.0297 (2.6), 215.0340 (2.4), 203.0343 (0.1), 187.0397 (1.3), 163.0026 (0.9), 159.0442 (0.5)	8.99	0.897	A, B
95.	apigenin-*O*-(*p*-coumaroyl)hexoside	$C_{30}H_{26}O_{12}$	577.1341	577.1361 (64.0), 431.0975 (1.1), 269.0456 (100), 225.0552 (1.6), 151.0024 (1.2), 163.0388 (1.0), 145.0282 (14.1), 119.0489 (2.1), 117.0332 (12.5), 107.0124 (2.3)	9.02	1.665	A, B
96.	apigenin-*O*-(*p*-coumaroylhexoside) isomer	$C_{30}H_{26}O_{12}$	577.1341	577.1362 (100), 431.0975 (13.5), 413.0878 (7.8), 269.0456 (72.3), 225.0553 (0.8), 151.0025 (0.5), 145.0282 (75.0), 119.0489 (6.3), 117.0332 (36.7), 107.0123 (1.8)	9.07	1.872	A, B
97.	quercetagetin-3,6,3′(4′)-trimethyl ether [b]	$C_{18}H_{16}O_8$	359.0772	359.0777 (100), 344.0539 (79.1), 329.0304 (40.9), 314.0076 (2.6), 301.0356 (10.5), 286.0121 (8.1), 258.0180 (2.8), 242.0219 (13.3), 230.0210 (2.7), 214.0270 (9.1), 165.9904 (0.7), 163.0026 (0.6), 109.9985 (0.4)	9.07	1.196	B
98.	isorhamnetin [a,b]	$C_{16}H_{12}O_7$	315.0512	315.0513 (100), 300.0277 (48.2), 271.0249 (3.7), 255.0299 (2.4), 243.0094 (2.1), 227.0343 (1.3), 215.0344 (0.9), 199.0396 (0.5), 151.0025 (8.3), 107.0124 (8.5)	9.11	0.870	A, B
99.	jaceosidin (6-hydroxyluteolin-6,3′-dimethyl ether) [a,b]	$C_{17}H_{14}O_7$	329.0677	329.0671 (90.5), 314.0437 (100), 299.0200 (23.9), 271.0250 (37.7), 243.0298 (4.4), 227.0352 (2.6), 215.0345 (1.7), 199.0394 (8.8), 165.9893 (1.2), 164.9815 (1.8), 163.0025 (0.8), 136.9864 (2.0), 133.0281 (5.5)	9.14	1.319	A, B
100.	naringenin *O*-(*p*-coumaroyl)hexoside	$C_{30}H_{28}O_{12}$	579.1497	579.1520 (100), 433.1131 (0.7), 415.1029 (2.4), 271.0614 (70.7), 227.0710 (0.7), 163.0389 (11.9), 151.0025 (35.4), 145.0282 (47.2), 119.0488 (34.0), 107.0124 (14.2)	9.15	1.241	A, B

101.	naringenin *O*-(*p*-coumaroylhexoside) isomer	$C_{30}H_{28}O_{12}$	579.1497	579.1517 (100), 271.0614 (72.7), 163.0390 (12.6), 151.0025 (41.3), 145.0283 (52.2), 119.0488 (39.7), 107.0124 (15.4)	9.25	0.871	A, B
102.	axillarin [b]	$C_{17}H_{14}O_8$	345.0616	345.0620 (100), 330.0386 (34.8), 315.0152 (2.5), 302.0435 (11.0), 287.0199 (34.4), 273.0413 (2.0), 259.0247 (9.6), 258.0170 (13.0), 243.0299 (2.0), 231.0298 (4.0), 215.0342 (4.8), 203.0342 (3.2), 165.9900 (2.0), 149.0233 (1.9), 139.0390 (0.3), 109.9996 (1.4)	9.25	1.042	A, B
103.	quercetin-7,3′(4′)-dimethyl ether [b]	$C_{17}H_{14}O_7$	329.0677	329.0671 (100), 314.0437 (88.5), 299.0201 (33.4), 271.0250 (61.1), 243.0297 (1.1), 227.0341 (0.5), 199.0388 (1.3), 161.0230 (0.4), 151.0021 (1.2), 107.0120 (0.4)	9.44	1.319	A, B
104.	cirsimaritin [b]	$C_{17}H_{14}O_6$	313.0719	313.0722 (100), 298.0483 (54.6), 283.0249 (50.5), 269.0458 (4.3), 255.0300 (14.3), 227.0352 (4.2), 163.0026 (11.9), 135.0073 (4.8), 117.0331 (10.6)	10.38	1.401	A, B
105.	eupatilin [a,b]	$C_{18}H_{16}O_7$	343.0812	343.0826 (100), 328.0591 (66.8), 313.0357 (32.2), 298.0122 (19.0), 285.0407 (5.3), 270.0171 (19.6), 242.0217 (3.8), 214.0263 (3.1), 198.0317 (2.4), 165.9898 (0.4), 164.9819 (1.3), 163.0022 (1.9), 147.0433 (2.0), 136.9862 (1.9), 132.0206 (2.2)	10.67	0.653	A, B
106.	ayanin [b]	$C_{18}H_{16}O_7$	343.0812	343.0825 (100), 328.0590 (38.6), 313.0357 (33.3), 299.0564 (2.5), 285.0405 (23.7), 270.0169 (13.6), 242.0220 (3.1), 214.0263 (1.3), 151.0021 (0.3)	11.03	0.390	A, B
107.	genkwanin [a,b]	$C_{16}H_{12}O_5$	283.0612	283.0612 (100), 268.0379 (54.67), 239.0351 (3.00), 151.0024 (1.94), 117.0331 (3.41)	11.59	-0.103	A, B
108.	ayanin isobar [b]	$C_{18}H_{16}O_7$	343.0812	343.0826 (100), 328.0591 (61.5), 313.0358 (40.4), 285.0408 (12.7), 257.0457 (6.2), 226.0268 (31.3), 214.0264 (1.7), 161.0233 (1.2)	11.76	0.653	A, B

[a] Identified by comparison with an authentic standard; [b] reported for the first time in *E. ritro*; A—flower head extract; B—leaf extract.
